# CT-Guided Transthoracic Biopsy of Lung Lesions Using a Non-Coaxial Biopsy Needle Technique: CT Characteristics Predictive for Diagnostic Accuracy and Pneumothorax

**DOI:** 10.5334/jbsr.2429

**Published:** 2021-06-30

**Authors:** Barbara Geeroms, Lesley Cockmartin, Johan Coolen, Adriana Dubbeldam, Johny Verschakelen, Ani Nikoghosyan, Walter De Wever

**Affiliations:** 1 AZ Maria Middelares, BE; 2 UZ Leuven, BE

**Keywords:** retrospective studies, computed tomography, lung biopsies, risk factors, pneumothorax

## Abstract

**Objectives:**

To analyze computed tomography (CT) characteristics predictive for diagnostic accuracy and pneumothorax in CT fluoroscopy-guided transthoracic biopsy (CTF-TTB) of lung lesions using non-coaxial biopsy needle technique.

**Methods:**

Retrospectively 274 lung lesion biopsies with confirmed histology were included in our study. CTF-TTB was done using an 18-gauge non-coaxial cutting needle. Diagnostic accuracy rates were calculated per lesion size and CT and procedural characteristics were evaluated for their predictive value regarding diagnostic accuracy and development of pneumothorax (maximal nodule diameter, distance to pleura, location per lung segment, nodule composition, benign versus malignant histology, and number of specimens).

**Results:**

Overall diagnostic accuracy of CTF-TTB was high (93%). Diagnostic accuracy for lesions ≤10 mm was 81%. Maximal nodule diameter was the only predictive CT characteristic for diagnostic success (p = 0.03). Pneumothorax occurred in 27%. Distance of lesion to pleura was the only risk factor for pneumothorax (p < 0.00001). Pneumothorax rates were significantly lower in subpleural lesions (14%) compared to those located 1–10 mm (47%), 10–20 mm (33%), and >20 mm from pleura (29%).

**Conclusions:**

High diagnostic accuracy rates were achieved with CTF-TTB using non-coaxial biopsy technique, even for lesions ≤10 mm. Pneumothorax rates were comparable with other studies. Lesion size was the only predictive CT characteristic for diagnostic accuracy. Distance to pleura was the only risk factor for pneumothorax.

## Introduction

Computed tomography (CT) guided transthoracic biopsies of lung lesions are routinely performed for diagnosis of lesions difficult to access with bronchoscopy, especially small and peripheral lung lesions [1].

The introduction of CT fluoroscopy-guided transthoracic biopsies (CTF-TTB) has resulted in significant improvement of diagnostic accuracy, with diagnostic accuracy rates from 94% to 98% (2–4) and remaining high (88% to 93%) for lesions ≤10 mm [2,5].

We sought to determine the diagnostic accuracy rates for these small lesions (≤10 mm) with the non-coaxial biopsy technique used in our institution and the CT characteristics of pulmonary lesions predictive for diagnostic accuracy and pneumothorax.

## Materials and Methods

**Figure 1:**
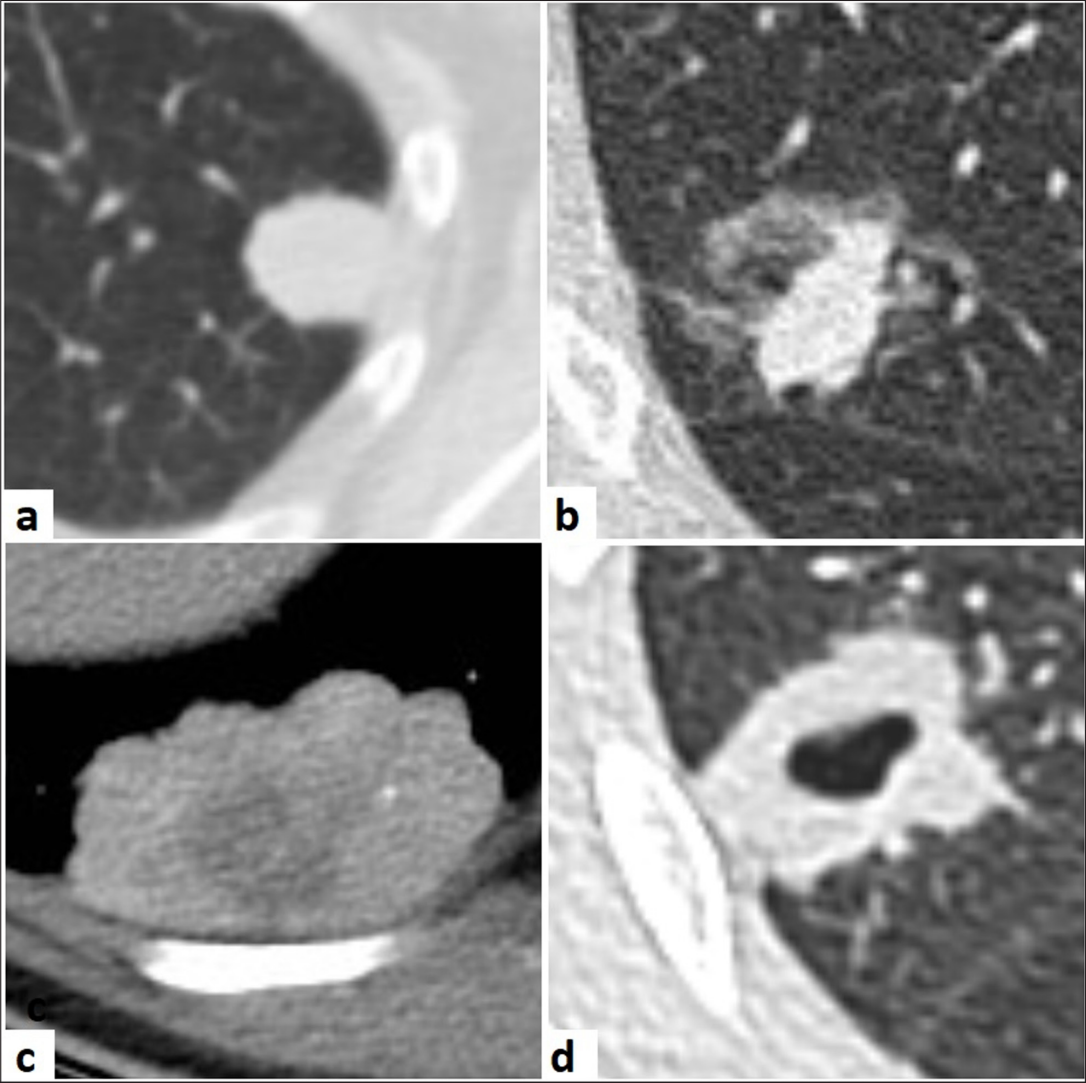
Examples of biopsied lesions: solid nodule (**a**), mixed solid-ground glass nodule (**b**), necrotic nodule (**c**), cavitated nodule (**d**).

From October 2016 to October 2018, 300 CT fluoroscopy-guided lung biopsies were performed at our institution. Our study population included 274 lesions of which the biopsy diagnosis was confirmed with definite pathology diagnosis, PET-CT or follow-up imaging. The following variables regarding the lesion, procedure and specimens were collected: maximal lesion diameter, lesion composition (solid, mixed solid-ground glass, necrotic, cavitated) (***Figure 1***), distance of lesion to pleura, localization per lung segment, number of biopsy specimens and occurrence of pneumothorax. Lesion measurements were done manually on diagnostic CT images before looking up biopsy diagnosis and final diagnosis.

True nature of a pulmonary lesion (benign or malignant) was confirmed with histological analysis of the surgically resected lesion or with follow-up imaging or PET-CT. A lesion was considered benign when it met one of following criteria: 1) stable at follow-up imaging at least six months after biopsy, 2) spontaneous regression or regression under antibiotics or corticosteroids (without chemotherapy, immune therapy, or radiation therapy) or 3) no enhancement on PET-CT and *not* clinically suspicious for malignancy. A lesion was considered malignant when it met one of following criteria: 1) doubling time between 20 and 100 days [6] or 2) very suspicious on PET-CT. Biopsy results were classified in three categories: malignant, benign and not representative. A biopsy result was not representative if the specimen contained no pathological tissue. A ‘true positive’ biopsy result was a malignant biopsy result, confirmed with histology of the resected specimen or nuclear or follow-up imaging. A ‘true negative’ biopsy result was a benign biopsy result confirmed the same way. ‘False positive’ and ‘false negative’ biopsy diagnoses were diagnoses of respectively malignant and benign lesions, contradicted on definite histology or follow-up/nuclear imaging. A biopsy was *diagnostically accurate* when biopsy diagnosis was confirmed by histological analysis of the resected lesion or by follow-up or nuclear imaging.

In total 26 lesions were excluded, because they were not resected and had no or uncertain character on PET-CT or follow-up imaging. The latter included: doubling time of <20 days or >100 days or lesion regression under chemotherapy or immunotherapy. Lesions with biopsy diagnosis of malignancy that underwent radiation therapy afterwards were considered malignant.

**Figure 2:**
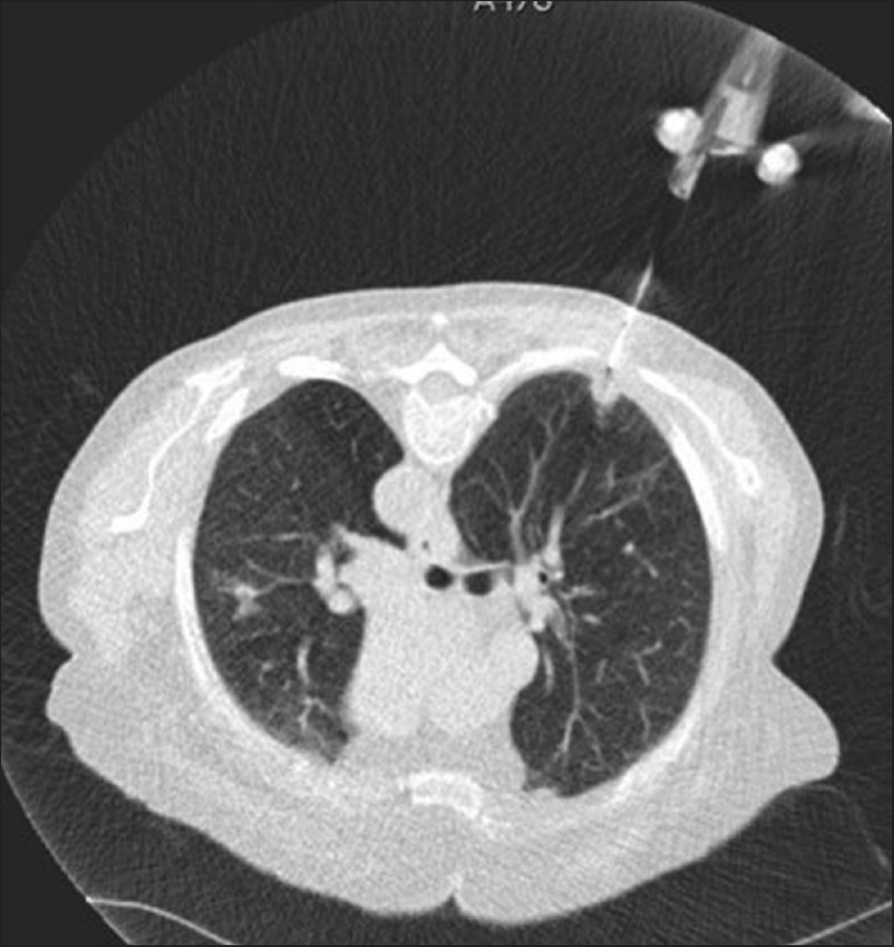
CT fluoroscopic image during a CT fluoroscopy guided transthoracic biopsy of a subpleural pulmonary lesion anteriorly in the left upper lobe.

All procedures were performed by a chest radiologist with 20 years of experience in CTF-TTB. Before each procedure, the diagnostic images were reevaluated to determine the best biopsy route and patient positioning. Patients were positioned supine, prone, or sideways, depending on lung lesion location and patient cooperation, and preferably positioned so the needle could track the shortest route from pleura to lesion and avoid fissures, bronchi, large vessels, and emphysema. Necrotic and cavitated areas in lung lesions were avoided if possible. In mixed solid-ground glass lesions the solid component was preferably biopsied. CT fluoroscopy guidance (Siemens-Flash) was used to visualize the lung lesion and guide the needle. CT fluoroscopic images were acquired with scanning parameters: 0.75s/rotation, tube voltage 100–120 kV; current 20 mA; collimation 5 mm. The biopsy was performed with 18-gauge non-coaxial semi-automated cutting needle (BARD, Max-Core) (***Figure 2***). CT fluoroscopy was intermittently performed to guide needle advancement but minimize radiation exposure. Pleura was punctured and the specimen was taken during one breath hold to optimize correct needle aiming and minimize pneumothorax risk.

The aim in each procedure was to obtain two representative specimens. In 32 cases only one specimen was obtained, because the patient wished to cease the procedure, significant pneumothorax had occurred after the first biopsy, or one good biopsy specimen had already been obtained in a patient with higher complication risk (e.g., severe emphysema). In 20 cases three specimens were obtained, because it was requested for determining molecular markers or because the radiologist found the first two specimens of insufficient quality. All patients underwent chest X-Ray four hours after the procedure to check for pneumothorax.

### Statistical analysis

Data analysis was performed using SPSS software (IBM-SPSS version 13.0, Chicago). Statistical comparisons between diagnostically accurate and not diagnostically accurate groups were performed using the Pearson Chi-square test for categorical variables (lung segment, lesion composition, number of biopsy specimens) and Mann-Whitney test for continuous variables (maximal lesion diameter and distance from lesion to pleura). Next, binary logistic regression analysis was performed to determine independent prognostic factors for diagnostic success. In the regression analysis, continuous variables (i.e., maximal lesion diameter and distance from lesion to pleura) were categorized into discrete groups. All tests were performed as two-sided at significance level α of 0.05.

## Results

### Diagnostic accuracy

***Table 1*** lists lesion and procedure characteristics, diagnostic success rates, and failure rates.

**Table 1:** Characteristics of 274 CT fluoroscopy-guided biopsies, diagnostic accuracy, and failure rates and P-values of univariate analysis of possible predictive factors.


CHARACTERISTIC	VALUE (N,%)	DIAGNOSTIC ACCURACY (N,%)	DIAGNOSTIC FAILURE (N,%)	P-VALUE

No. Patients	274			

Nodule size (mm)				0.03

Mean ± SD	28 ± 18			

Range	4–11			

≤10 mm	31 (11%)	25 (81%)	6 (19%)	

11–20 mm	91 (33%)	85 (93%)	6 (7%)	

>20 mm	152 (55%)	145 (95%)	7 (5%)	

Nodule location (n,%)				0.91

Upper lobes	132 (48%)	123 (93%)	9 (7%)	

Middle lobe/lingula	22 (8%)	20 (91%)	2 (9%)	

Lower lobes	120 (44%)	112 (93%)	8 (7%)	

Nodule consistency (n,%)				0.91

Solid	189 (69%)	174 (92%)	15 (8%)	

Mixed solid – ground glass	26 (9%)	26 (100%)	0	

Necrosis	32 (12%)	30 (94%)	2 (6%)	

Excavation	27 (10%)	25 §93%)	2 (7%)	

Distance to pleura (mm)				0.48

Mean ± SD	6 ± 9			

Range	0–50			

0 mm	129 (47%)	119 (92%)	10 (8%)	

≤10 mm	73 (27%)	69 (94%)	4 (6%)	

11–20 mm	48 (18%)	44 (92%)	4 (8%)	

>20 mm	24 (9%)	23 (96%)	1 (4%)	

No. Specimens				0.14

Mean ± SD	2 ± 0,4			

Range	1–3			

1	32 (12%)	27 (84%)	5 (16%)	

2	222 (81%)	209 (94%)	13 (6%)	

3	20 (7%)	19 (95%)	1 (5%)	

Final diagnosis (n,%)				0.36

Benign	67 (24%)	64 (96%)	3 (4%)	

Malignant	207 (76%)	191 (92%)	16 (8%)	


Diagnostic accuracy rates of CTF-TTB per lesion size are shown in ***Table 2***. Overall diagnostic accuracy was 255/274 (93%); diagnostic accuracy for lesions ≤10 mm was 25/31 (81%).

**Table 2:** Diagnostic accuracy, sensitivity, specificity, negative predictive value (NPV), and positive predictive value (PPV) of CT fluoroscopy-guided lung biopsy for 274 lesions according to lesion size.


	≤10 MM (N = 31)	11–20 MM (N = 91)	21–30 MM (N = 66)	>30 MM (N = 86)	OVERALL (N = 274)

True-positive results	48% (n = 16)	74% (n = 67)	68% (n = 45)	73% (n = 63)	69% (n = 191)

True-negative results	32% (n = 9)	20% (n = 18)	26% (n = 17)	23% (n = 20)	24% (n = 64)

False-positive results	0	0	0	0	0

False-negative results	3% (n = 1)	1% (n = 1)	2% (n = 1)	3% (n = 3)	2% (n = 6)

Not representative results	16% (n = 5)	5% (n = 5)	5% (n = 3)	0	5% (n = 13)

**Diagnostic accuracy**	**81%**	**93%**	**94%**	**97%**	**93%**

Sensitivity	76%	94%	92%	95%	92%

Specificity	100%	100%	100%	100%	100%

NPV	67%	83%	81%	87%	81%

PPV	100%	100%	100%	100%	100%


Biopsy was not representative in 5% (n = 13) of cases. Ten of these were malignant and three were benign; these were included as respectively false-negative and true-negative lesions in the calculation of sensitivity, specificity, negative predictive value (NPV) and positive predictive value (PPV). Ten of the not representative biopsies were lesions ≤20 mm. In four not representative biopsies only one tissue sample was taken.

There were six false-negative biopsies (***Table 3***): three were lesions >40 mm.

**Table 3:** False-negative biopsies: lesion size, biopsy diagnosis and final diagnosis.


LESION SIZE (MM)	BIOPSY DIAGNOSIS	FINAL DIAGNOSIS

8	fibrosis, inflammation, squamous cells; differential diagnosis: reactive squamous metaplasia or squamous cell carcinoma	/*

15	fibrosis, inflammation	metastasis of thyroid cell carcinoma

23	inflammation	PEComa

47	fibrosis	adenocarcinoma

54	organizing pneumonia with necrosis	adenocarcinoma

84	inflammation, fibrosis, residual epithelial structures that could be tumor nests	squamous cell carcinoma


* No final histology available, malignant on follow-up imaging.

A malignant biopsy diagnosis was made in 191 cases; the real number of malignant lesions was 207 (43 confirmed with histology of the resected lesion, 5 confirmed on follow-up imaging and 159 on PET-CT).

Diagnosis of benign lesion was made in 70 cases on biopsy; the real number of benign lesions was 67 (7 confirmed with histology of the resected lesion, 57 confirmed on follow-up imaging, and 3 on PET-CT).

Pathology diagnoses and their frequencies are listed in ***Table 4***.

**Table 4:** Overview of the histopathology diagnoses, their frequencies, and diagnostic accuracy rates.


HISTOPATHOLOGY DIAGNOSIS	PROPORTION OF LESIONS (N,%)	DIAGNOSTIC ACCURACY (%)

**Malignant lesions**	207 (76%)	93% (191/207)

NSCLC		

Adenocarcinoma	88 (32%)	97% (85/88)

Squamous cell carcinoma	38 (14%)	92% (35/38)

Large cell carcinoma	7 (3%)	100% (7/7)

Carcinoid	3 (1%)	100% (3/3)

NSCLC, NOS	5 (2%)	100% (5/5)

SCLC	10 (4%)	100% (10/10)

Metastatis from extrathoracic malignancy	35 (13%)	80% (28/35)

Lymphoma	11 (4%)	100% (11/11)

Other *^1^	10 (4%)	70% (7/10)

**Benign lesions**	67 (24%)	96% (64/67)

Organizing pneumonia	14 (5%)	93% (13/14)

Infection	3 (1%)	100% (3/3)

Aspecific inflammation *^2^	41 (15%)	98% (40/41)

Other *^3^	9 (3%)	89% (8/9)


*^1^ Other malignant lesions: adenosquamous carcinoma (n = 2), mixed NSCL-SCLC (n = 1), PEComa (n = 1), sarcoma (n = 2), mesothelioma (n = 1), muco-epidermoid tumor (n = 1), no definite (correct) histology – proven malignant on FU (n = 2). *^2^ A non-defined percentage of the biopsies that showed aspecific inflammation appeared to be infection afterwards. *^3^ Other benign lesions: sarcoidosis (n = 1), hamartoma (n = 2), Wegener (n = 2), benign mesenchymal tumor (n = 1), intrapulmonary lymph node (n = 2), no histology – proven benign on follow-up imaging (n = 1).

### Predictive factors for diagnostic accuracy

Univariate analysis showed only lesion size as independent predictive factor for diagnostic accuracy (p = 0.03) (***Table 1***). Other parameters, such as number of specimens (p = 0.14), lesion composition (p = 0.91), location per lung segment (p = 0.91) and final diagnosis (benign versus malignant) (p = 0.36) were no significant predictive factors for diagnostic accuracy.

Maximal lesion diameters were divided in three groups: ≤10 mm, 10–20 mm, and >20 mm. Univariate binary logistic regression showed that nodules >20 mm were four times (=OR) (p = 0.02) more likely to result in accurate biopsy diagnosis than nodules ≤10 mm. No significant difference in diagnostic accuracy was found for nodules 10–20 mm versus nodules ≤10 mm (OR = 3.1, p = 0.08).

To analyze possible effect of distance of lesion to pleura on diagnostic accuracy, lesions were divided into four groups: immediately subpleural, distance ≤10 mm, 10–20 mm, and >20 mm. There were no significant differences in diagnostic accuracy between these groups (p = 0.86). Subanalyses were performed based on maximal lesion diameters: possible effects of distance to pleura on diagnostic accuracy were investigated for lesions ≤20 mm and lesions ≤10 mm. No statistically significant effect of distance to pleura was observed in these subgroups (p = 0.99 for lesions ≤20 mm; p = 0.74 for lesions ≤10 mm).

Although the number of biopsy specimens was no significant predictive factor for diagnostic success (p = 0.14), there was tendency towards higher diagnostic success when two or three biopsy specimens were taken (respectively 95% and 94% diagnostic success) compared to one specimen (84% diagnostic success).

### Pneumothorax

Pneumothorax occurred in 27% (n = 75) of procedures. In most cases (n = 61) pneumothorax was already present on CT during the procedure; in 14 cases pneumothorax was seen on chest X-ray four hours after the procedure. A chest tube had to be inserted in six patients (2% of all study patients).

Distance of lesion to pleura appeared to be a statistically significant risk factor for pneumothorax (p < 0.00001). In lesions located immediately subpleural, 14% developed a pneumothorax. This percentage was significantly lower compared to lesions located 1–10 mm (47%), 10–20 mm (33%), and >20 mm from pleura (29%).

Neither number of specimens (p = 0.12) nor cavitation (p = 0.86) were risk factors for pneumothorax.

## Discussion

### Diagnostic accuracy

Most centers use a coaxial needle technique in CTF-TTB, because of a presumed higher diagnostic accuracy and lower complication rates [2,3]. However, in their comparative study, Wu et al. already showed high diagnostic accuracy, up to 98%, using coaxial *and* single-needle techniques. Our study evaluated non-coaxial 18-gauge needle biopsy technique and found overall diagnostic accuracy of 93%, comparable with other studies (***Table 5***). Five of the thirteen not representative samples were lesions ≤10 mm; there were no unrepresentative samples in lesions >30 mm. Chances of missing the target are higher in smaller targets. As a result, there is a larger variability on the accuracy for lesions ≤10 mm across the literature (***Table 5***).

**Table 5:** Diagnostic accuracy rates and complication rates of CT-guided lung biopsies in the literature.


STUDIES WITH USED BIOPSY TECHNIQUE	OVERALL DIAGNOSTIC ACCURACY OF CT-GUIDED LUNG BIOPSY (%)	DIAGNOSTIC ACCURACY FOR LESIONS ≤10 MM (%)	PNEUMOTHORAX RATE (%)

Geeroms et al.	93	81	27

Ahn et al. [7] 19 G outer coaxial needle; no CT fluoroscopy	97		27

Wu et al. [3] 17 G outer coaxial needle and 18 G single needle technique; no CT fluoroscopy	98		

Hiraki et al. [2] 19 G outer needle coaxial technique with CT fluoroscopy	95	93	43

Yeow et al. [8] 16, 18 or 20 G outer coaxial needle; no CT fluoroscopy	95	84	

Choi et al. [2] 20 G outer coaxial needle; no CT fluoroscopy	95		19

Gupta et al. [4] 18 G outer coaxial needle with CT fluorscopy	94		22

Césara et al. [9]	91		21

Heyer et al. [10] 16 G single needle technique; no CT fluoroscopy	89		26

Tsukada et al. [11] 18 G outer coaxial needle; no CT fluoroscopy	83	67	22

Oliveira et al. [12]		87	29

Takuji et al. [5] 20 G single needle with CT fluoroscopy		88	35

Chang et al. [13] 17 G outer coaxial needle; no CT fluoroscopy		87	

Elshafee et al. [16] 18 G single needle technique; no CT fluoroscopy			44

Montaudon et al. [14] 19 G outer coaxial needle technique; no CT fluoroscopy			17

Shiekh et al. [18] 17 or 19 G outer coaxial needle technique; no CT fluoroscopy			25


We registered six false-negative biopsies including three lesions ≥40 mm. Small size was not predictive for false-negative biopsy results. There was tendency towards a higher likelihood of false-negative results in lesions with significant fibrosis or necrosis (four out of six false-negative lesions had significant fibrosis on pathology and one showed necrosis). Indeed, some lesions appeared large on CT because of extensive fibrosis or necrosis. CT fluoroscopy was done without intravenous contrast medium, so necrosis or fibrosis could often not be avoided during biopsy. In two false-negative biopsies the pathologist mentioned possible malignant cells but found the samples insufficient for definite diagnosis. In both cases, only two biopsy specimens were obtained, a third specimen may have aided in diagnosis of malignancy.

### Predictive factors for diagnostic accuracy

Lesion size was the only significant predictive factor for diagnostic success. Diagnostic accuracy decreased significantly in smaller lesions. However, even in lesions ≤10 mm, diagnostic accuracy was still high, at 81%, so it was still acceptable to perform CTF-TTB in these lesions (***Table 5***).

The sample size of our study probably prevented a more in-depth analysis of predictive factors for accuracy: first, the number of lesions ≤5 mm was too small to evaluate diagnostic accuracy in these lesions. Second, although half of our false-negative biopsies (3/6) were in lesions >30 mm due to fibrosis and/or necrosis, lesion size of >40 mm was not a significant (p = 0.98) risk factor for diagnostic failure as earlier reported by Hiraki et al. [2]. Third, despite the number of specimens was not a significant predictive factor for diagnostic success (p = 0.14), we must avoid taking only one biopsy specimen, as diagnostic accuracy in this group was at least 10% lower compared to the groups with ≥2 specimens. Other studies confirm that acquisition of more specimens increases diagnostic accuracy, because sampling error decreased [2,9]. We routinely obtained only two biopsy specimens to minimize pneumothorax risk. One would suspect that pneumothorax risk increases when the pleura is punctured more frequently and, in a non-coaxial needle technique this correlates with number of specimens. However, number of specimens was not a significant risk factor for pneumothorax.

There was no significant correlation between distance of lesion to pleura and diagnostic accuracy. Lesion composition was also not a predictive factor for diagnostic success as previously reported by de Oliveira et al. [10]. Lastly, other studies described benign histology [11] and location in lower lobes [2,10] as risk factors for diagnostic failure. We couldn’t confirm this (***Table 4***).

### Pneumothorax

In our population 27% developed pneumothorax, which is comparable with literature (***Table 5***). One might think pneumothorax risk is correlated with number of pleural introductions, which is higher in single-needle technique compared to coaxial technique. However, Wu already showed that pneumothorax risk is not higher with a single-needle technique [3]. Our study confirmed their finding: number of needle introductions (which equals number of specimens) was not a significant risk factor for pneumothorax.

Pneumothorax risk was significantly lower in subpleural lesions compared to lesions at distance from pleura, which is intuitive, as no lung tissue is present between pleura and lesion. This was previously reported by Shiekh et al. (12). In our study pneumothorax risk decreased as distance between lesion and pleura increased. This is not in line with results of other studies [12,13,14,15,16].

Other authors found additional pneumothorax risk factors that were not confirmed in our study: right middle lobe location [13], subsolid lesions [13], number of pleural passages [10,13,15], small lesion size [12,13,14,15,16] and cavitation [14].

Risk of severe pneumothorax requiring chest tube placement was lower in our population (8% of all patients with pneumothorax) compared to most other studies (varying between 18% [15], 14% [13] and 11% [5]). Only Montaudon et al. had pneumothorax rate of 17%, of which only 3% required chest tube [8]. It is likely that the single-breath-hold technique during our procedure can explain our low incidence of (especially severe) pneumothorax. This can also explain why pneumothorax risk is not higher in our non-coaxial needle technique compared to coaxial needle techniques: the pleura is punctured multiple times in non-coaxial needle biopsy technique, but there is more friction of needle with pleura in coaxial needle technique, as the needle is kept into place during breathing.

## Conclusion

Small lesion size (≤10 mm) is a significant risk factor for diagnostic failure. Diagnostic accuracy in these small lesions is >80%, so they remain a good indication for CTF-TTB.

We achieved high diagnostic accuracy rates and low pneumothorax rates using the single-needle technique compared to other studies using coaxial needle technique. The only risk factor for pneumothorax was distance of lesion to pleura. Immediate subpleural located lesions had the lowest pneumothorax risk.
